# Supplementary material for: Hybrid Models and Biological Model Reduction with PyDSTool
Source: PLoS Comput Biol. 2012 Aug 9;8(8):e1002628. doi: 10.1371/journal.pcbi.1002628 (PMC3415397; doi:10.1371/journal.pcbi.1002628)
Supplement: Text S4 — Complete source code for the PyDSTool package (version 0.88.120504). Includes API documentation and help files linking to web pages. This file is identical to the current public release on Sourceforge.net. (ZIP) [file pcbi.1002628.s004.zip › PyDSTool/html/PyDSTool.Toolbox.optimizers.optimizer.standard_optimizer_modifying-module.html]

xml version="1.0" encoding="ascii"?


PyDSTool.Toolbox.optimizers.optimizer.standard\_optimizer\_modifying


| Home | Trees | Indices | Help | PyDSTool |
| --- | --- | --- | --- | --- |

Package PyDSTool ::
Package Toolbox ::
Package optimizers ::
Package optimizer ::
Module standard\_optimizer\_modifying

[hide private]

[frames] | no frames]

# Module standard\_optimizer\_modifying

source code

A standard optimizer with a special object that modifies the resulting
set of parameters


|  |  |
| --- | --- |
| Classes | [hide private] |

StandardOptimizerModifying  
A standard optimizer, takes a step and finds the best candidate
Must give in self.optimalPoint the optimal point after optimization
After each iteration the resulting optimization point is modified
by a call to a function

| Home | Trees | Indices | Help | PyDSTool |
| --- | --- | --- | --- | --- |

|  |  |
| --- | --- |
| Generated by Epydoc 3.0.1 on Fri May 4 15:24:05 2012 | http://epydoc.sourceforge.net |
